# Outcomes of laboratory‐confirmed SARS‐CoV‐2 infection in the Omicron‐driven fourth wave compared with previous waves in the Western Cape Province, South Africa

**DOI:** 10.1111/tmi.13752

**Published:** 2022-05-10

**Authors:** Mary‐Ann Davies, Reshma Kassanjee, Petro Rousseau, Erna Morden, Leigh Johnson, Wesley Solomon, Nei‐Yuan Hsiao, Hannah Hussey, Graeme Meintjes, Masudah Paleker, Theuns Jacobs, Peter Raubenheimer, Alexa Heekes, Pierre Dane, Jamy‐Lee Bam, Mariette Smith, Wolfgang Preiser, David Pienaar, Marc Mendelson, Jonathan Naude, Neshaad Schrueder, Ayanda Mnguni, Sue Le Roux, Kathleen Murie, Hans Prozesky, Hassan Mahomed, Liezel Rossouw, Sean Wasserman, Deborah Maughan, Linda Boloko, Barry Smith, Jantjie Taljaard, Greg Symons, Ntobeko A. B. Ntusi, Arifa Parker, Nicole Wolter, Waasila Jassat, Cheryl Cohen, Richard Lessells, Robert J. Wilkinson, Juanita Arendse, Saadiq Kariem, Melvin Moodley, Milani Wolmarans, Keith Cloete, Andrew Boulle, Western Cape and South African National Departments of Health in collaboration with the National Institute for Communicable Diseases in South Africa Affiliations

**Affiliations:** ^1^ Health Intelligence, Western Cape Government: Health Cape Town South Africa; ^2^ Centre for Infectious Disease Epidemiology and Research, School of Public Health and Family Medicine, University of Cape Town Cape Town South Africa; ^3^ Division of Public Health Medicine School of Public Health and Family Medicine, University of Cape Town Cape Town South Africa; ^4^ National Department of Health Pretoria South Africa; ^5^ Division of Medical Virology University of Cape Town Cape Town South Africa; ^6^ National Health Laboratory Service Cape Town South Africa; ^7^ Groote Schuur Hospital, Western Cape Government: Health Cape Town South Africa; ^8^ Department of Medicine University of Cape Town Cape Town South Africa; ^9^ Wellcome Centre for Infectious Disease Research in Africa, Institute of Infectious Disease and Molecular Medicine, University of Cape Town Cape Town South Africa; ^10^ Division of Health Systems and Public Health, Department of Global Health Stellenbosch University Tygerberg South Africa; ^11^ Division of Medical Virology Stellenbosch University Stellenbosch South Africa; ^12^ Rural Health Services, Western Cape Government: Health Cape Town South Africa; ^13^ Division of Infectious Diseases and HIV Medicine, Department of Medicine University of Cape Town Cape Town South Africa; ^14^ Mitchells Plain Hospital, Western Cape Government: Health Cape Town South Africa; ^15^ Tygerberg Hospital, Western Cape Government: Health Parow South Africa; ^16^ Department of Medicine Stellenbosch University Stellenbosch South Africa; ^17^ Khayelitsha District Hospital, Western Cape Government: Health Khayelitsha South Africa; ^18^ Karl Bremer Hospital, Western Cape Government: Health Bellville South Africa; ^19^ Western Cape Government: Health Cape Town South Africa; ^20^ Metro Health Services, Western Cape Government: Health Cape Town South Africa; ^21^ Division of Infectious Diseases, Department of Medicine Stellenbosch University Tygerberg South Africa; ^22^ National Institute for Communicable Diseases, National Health Laboratory Service Johannesburg South Africa; ^23^ Faculty of Health Sciences, School of Public Health, University of the Witwatersrand Johannesburg South Africa; ^24^ KwaZulu‐Natal Research, Innovation & Sequencing Platform, University of KwaZulu‐Natal Durban South Africa; ^25^ Francis Crick Institute London UK; ^26^ Department of Infectious Diseases Imperial College London London UK

**Keywords:** COVID‐19, Delta, immunity, omicron, prior infection, sub‐Saharan Africa, vaccination

## Abstract

**OBJECTIVES:**

The objective was to compare COVID‐19 outcomes in the Omicron‐driven fourth wave with prior waves in the Western Cape, assess the contribution of undiagnosed prior infection to differences in outcomes in a context of high seroprevalence due to prior infection and determine whether protection against severe disease conferred by prior infection and/or vaccination was maintained.

**METHODS:**

In this cohort study, we included public sector patients aged ≥20 years with a laboratory‐confirmed COVID‐19 diagnosis between 14 November and 11 December 2021 (wave four) and equivalent prior wave periods. We compared the risk between waves of the following outcomes using Cox regression: death, severe hospitalisation or death and any hospitalisation or death (all ≤14 days after diagnosis) adjusted for age, sex, comorbidities, geography, vaccination and prior infection.

**RESULTS:**

We included 5144 patients from wave four and 11,609 from prior waves. The risk of all outcomes was lower in wave four compared to the Delta‐driven wave three (adjusted hazard ratio (aHR) [95% confidence interval (CI)] for death 0.27 [0.19; 0.38]. Risk reduction was lower when adjusting for vaccination and prior diagnosed infection (aHR: 0.41, 95% CI: 0.29; 0.59) and reduced further when accounting for unascertained prior infections (aHR: 0.72). Vaccine protection was maintained in wave four (aHR for outcome of death: 0.24; 95% CI: 0.10; 0.58).

**CONCLUSIONS:**

In the Omicron‐driven wave, severe COVID‐19 outcomes were reduced mostly due to protection conferred by prior infection and/or vaccination, but intrinsically reduced virulence may account for a modest reduction in risk of severe hospitalisation or death compared to the Delta‐driven wave.

## INTRODUCTION

Following the identification and early spread of the Omicron (B.1.529) SARS‐CoV‐2 variant of concern (VOC) in November 2021, South Africa observed the steepest surge in COVID‐19 cases to date [1, 2]. With more than 50 mutations across its genome, in vitro, ex vivo and modelling studies have uncovered potential changes in the biology of Omicron compared to previous VOCs, such as tropism, immune escape and improved transmissibility [3, 4, 5, 6, 7, 8].

**FIGURE 1:**
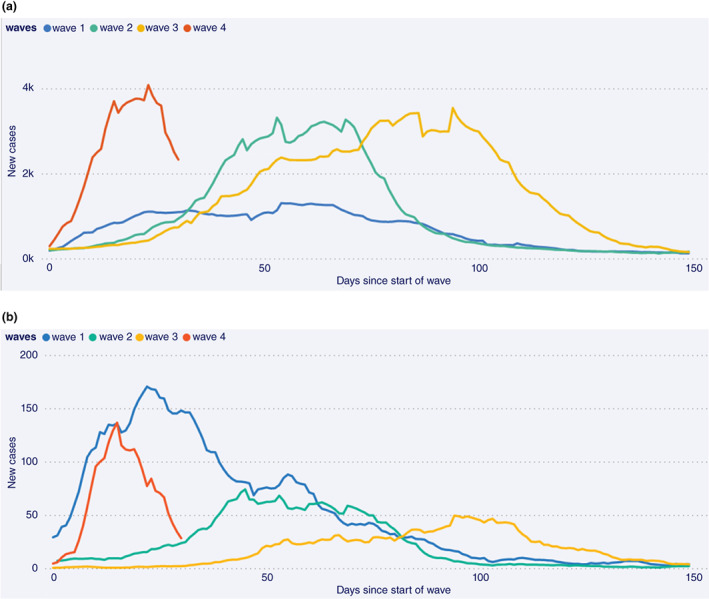
Daily new cases (7‐day moving average) by days since the start of each wave in (a) the Western Cape Province and (b) Khayelitsha subdistrict, Cape Town, South Africa from 10 March 2020 to 31 December 2021

South Africa had previously experienced three COVID‐19 waves related to different SARS‐CoV‐2 variants (ancestral strain, Beta and Delta, respectively), each more clinically severe than the previous one with substantial mortality [9, 10]. These waves have resulted in high seroprevalence of ~70% from prior infection, especially in poorer communities where social distancing is challenging [11, 12]. Whilst such high seroprevalence came at the cost of exceptionally high mortality during the first wave of COVID‐19, these areas were relatively protected from both infections and severe disease in subsequent waves [13]. For example, in the large urban township of Khayelitsha, the poorest subdistrict in Cape Town, anti‐nucleocapsid antibody seroprevalence resulting from prior infection was 45% by the end of wave one [11], increasing to >70% by the end of wave three (Nei‐Yuan Hsiao, personal communication). Unlike the relatively small and slow waves two and three in Khayelitsha (compared to large steep waves in the rest of the province), evidence of immune escape from infection with Omicron is illustrated by the steep rise in cases in Khayelitsha in wave four, similar to that of wave one, in comparison to waves two and three (Figure 1).

Whilst there is emerging biological evidence of possible lower virulence of Omicron compared to previous variants due to modified cell entry mechanisms and preferential replication in bronchi rather than the lung parenchyma [6, 7, 14, 15], even a virus resulting in similar or lower clinical severity to earlier variants could overwhelm health services if protection conferred by prior COVID‐19 infection and/or SARS‐CoV‐2 vaccination against severe disease is reduced. Whilst there are now several reports from South Africa and other countries of reduced risk of severe disease in the fourth wave and in patients infected with Omicron compared to Delta [16, 17, 18, 19, 20, 21] in the context of a high seroprevalence setting such as South Africa, with moderate SARS‐CoV‐2 vaccination coverage (39% and 46% of adults fully vaccinated in South Africa and the Western Cape, respectively, by end December 2021) [22], it is important to establish whether protection against severe disease conferred by prior infection and/or vaccination is maintained against Omicron. Furthermore, to what extent does such protection account for milder clinical presentation of Omicron cases versus inherent differences in virulence of Omicron itself compared to previous variants? Such comparisons should not be limited to Delta, which was itself more severe than previous variants [23, 24, 25, 26] and should fully account for the increased proportion of reinfections in an immune escape variant such as Omicron compared to other variants [18, 27]. Whilst Omicron's immune evasion allows for vaccine breakthroughs and reinfections, if protection against severe disease conferred by prior infection and/or vaccination is maintained, population data should reflect milder clinical illness due to a higher proportion of Omicron cases with reinfections/breakthroughs compared to cases of variants without escape from immunity against infection [18, 27].

We compared outcomes of laboratory‐confirmed SARS‐CoV‐2 infections across four successive waves in those aged ≥20 years using public sector services in the Western Cape Province, South Africa, accounting for prior infection and vaccination. We also assessed whether protection against severe disease conferred by prior diagnosed infections and/or vaccination was maintained in those infected during the Omicron‐driven fourth wave and examined the extent to which undiagnosed prior infection may account for observed reductions in clinical severity during the Omicron wave.

## METHODS

### Study design

We conducted a cohort study using de‐identified data from the Western Cape Provincial Health Data Centre (WCPHDC) of public sector patients aged ≥20 years with a laboratory‐confirmed COVID‐19 diagnosis (positive SARS‐CoV‐2 PCR or antigen test). For this analysis, each wave was deemed to commence on the date when COVID‐19 hospital admissions in public sector patients showed a sustained >10% week‐on‐week increase. We included cases diagnosed from 7 days before the wave start (to account for the lag between infection/first symptoms and hospitalisation) and for the following 4 weeks, to allow for at least 2 weeks of follow‐up in the most recently diagnosed patients in wave four. We, thus, included data for the fourth wave on cases from 14 November to 11 December 2021, with follow‐up through to 26 December 2021, which corresponds to the period when Omicron rapidly became the dominant variant in the province accounting for nearly 100% of sequenced cases. [2] Database closure was 10 days later to allow for death reporting delays. For the first wave, since there were no prior admissions, we selected a period where case incidence was the same as that at the start of wave four. We included all eligible patients in the relevant wave periods in the analysis.

The study was approved by the University of Cape Town and Stellenbosch University Health Research Ethics Committees and Western Cape Government: Health. Individual informed consent requirement was waived for this secondary analysis of de‐identified data.

### Study population and data sources

The Western Cape has nearly 7 million inhabitants, of whom approximately 75% use public sector health services [28]. The WCPHDC has been described in detail [10, 29]. Briefly, WCPHDC consolidates administrative, laboratory and pharmacy data from routine electronic clinical information systems used in all public sector health facilities with linkage through a unique identifier. Multiple data sources are triangulated to enumerate health conditions such as diabetes mellitus (‘diabetes’), hypertension, tuberculosis and HIV‐1. Hospitalisations (private and public) and reported deaths with a positive SARS‐CoV‐2 laboratory test are recorded and reviewed daily. For patients with recorded South African national identity numbers, data are linked to the South African vital registry to identify deaths not recorded in the WCPHDC. SARS‐CoV‐2 vaccination commenced on 17 February 2021 for health workers and 17 May 2021 for the general population in age cohorts, starting with those aged ≥60 years and progressively expanding to younger ages. By 20 October 2021, vaccination was available to all individuals aged ≥12 years. All SARS‐CoV‐2 vaccinations administered in the country are recorded on the Electronic Vaccine Data System (EVDS). Vaccination data (dates and types of all SARS‐CoV‐2 vaccines given at any facility in the province) were obtained using the South African national identifier to link WPHDC data to EVDS data for a specific individual.

### Statistical analysis

We assessed three outcomes: (i) death, (ii) severe hospitalisation or death and (iii) any hospitalisation or death. We only included outcomes within 14 days of COVID‐19 diagnosis to allow for comparable ascertainment across all wave periods. Hospitalisation included admission within 14 days before or after a COVID‐19 diagnosis except for admissions to long‐term psychiatric or rehabilitation facilities where the COVID‐19 diagnosis was likely to be incidental. Severe hospitalisation was defined as admission to intensive care or mechanical ventilation or oral/intravenous steroid prescription. Deaths within 14 days of COVID‐19 diagnosis were included unless a clear non‐COVID‐19 cause of death was recorded.

$$True\;HR_{Omicron:Delta}=\frac{Observed\;{\mathrm{HR}}_{Omicron:Delta}\left(1-\left(1-\gamma \right)\left(1-\rho \right)\left(\theta_{Delta}/\rho \right)\right)\;}{\left(1-\left(1-\gamma \right)\left(1-\rho \right)\left(\theta_{Omicron}/\rho \right)\right)}$$

We used Cox regression adjusted for age, sex, geographic location, comorbidities (diabetes mellitus, hypertension, chronic kidney disease, chronic pulmonary disease/asthma, previous/current tuberculosis and HIV), SARS‐CoV‐2 vaccination and prior diagnosed infection to assess differences in COVID‐19 outcomes between waves. Vaccination at the time of COVID‐19 diagnosis was defined as ‘fully’ (≥28 days post single dose vaccination with Janssen/Johnson & Johnson [Ad26.COV2.S] or ≥14 days post second dose of Pfizer–BioNTech [BNT162b2]) or ‘partially’ (≥21 days after single dose Ad26.COV2.S vaccine or first BNT162b2 dose until meeting criteria for fully vaccinated). Additional booster doses were not considered as these were not widely available except to health‐care workers and the severely immune compromised. Prior diagnosed infection was defined as ≥1 laboratory‐confirmed SARS‐CoV‐2 diagnosis ≥90 days previously without an intervening positive test. We used the approach described by Ferguson et al. [18] to assess the extent to which reductions in disease severity during the Omicron period may be attenuated by more unascertained prior infections in those with Omicron compared to patients infected with previous variants. As a base scenario, we assumed that immunity conferred by prior infection reduces the risk of death, severe hospitalisation or death and hospitalisation or death by 80%, 80% and 70%, respectively, and that only 15% of prior diagnosed infections were ascertained based on seroprevalence and excess death data [12, 30]. We then calculated a corrected hazard ratio for wave four versus wave three using the formula:

$$\text{True}\;{\mathrm{HR}}_{\text{Omicron}:\text{Delta}}=.$$

where *γ* is the relative risk of the outcome in those with versus those without prior infection, *ρ* is the proportion of reinfections detected and *θ* is the observed proportion of reinfections with Delta and Omicron, respectively [18]. The proportional‐hazard assumption (assessed with Schoenfeld residuals) [31] was violated for the effect of wave on the outcome of any hospitalisation or death only, as the hazards converged over time. We, therefore, also show the results of logistic regression for all of the outcomes which were very similar.

In addition, to assess whether protection conferred by prior infection and vaccination was similar in the Omicron and Delta periods, we compared the association between these variables and the three severe COVID‐19 outcomes separately for just the wave three and wave four periods. The wave three period was cases diagnosed between 1 September and 15 October 2021 as too few people had been vaccinated during early wave three (26 May to 23 June 2021) since vaccination only commenced for those 60 years and older on 17 May 2021. All analyses were conducted using Stata 17.1.

## RESULTS

We included 5144 patients diagnosed in wave four and 4403, 3902 and 3304 patients from waves three, two and one, respectively (Table 1). There was a greater proportion of patients aged 20–39 years in wave four (64%) compared to waves two (49%) and three (44%). The prevalence of comorbidities was mostly similar across waves, except for HIV‐1 which had the highest prevalence in wave one, decreased prevalence in waves two and three, but the increased prevalence in wave four (Table 1). The proportion with prior diagnosed infection was substantially higher in wave four (11%) compared to waves three (3.2%) or two (1.9%). In wave four, 38% and 5% of all COVID‐19 cases were fully or partially vaccinated, respectively.

**TABLE 1:** Characteristics and outcomes of COVID‐19 cases included from each of the four waves in the Western Cape Province, South Africa

	Wave 19 April to 7 May 2020^a^(*n* = 3304)	Wave 225 October to 21 November 2020^a^(*n* = 3902)	Wave 326 May to 23 June 2021^a^(*n* = 4403)	Wave 414 November to 11 December 2021^a^(*n* = 5144)
Male sex	889 (26.9%)	1376 (35.3%)	1638 (37.2%)	1737 (33.8%)
Age (years)				
20–39	2034 (61.6%)	1915 (49.0%)	1923 (43.7%)	3318 (64.5%)
40–49	666 (20.2%)	767 (19.7%)	847 (19.2%)	851 (16.5%)
50–59	391 (11.8%)	624 (16.0%)	787 (17.9%)	571 (11.1%)
60–69	144 (4.4%)	360 (9.2%)	472 (10.7%)	266 (5.2%)
≥70	69 (2.1%)	236 (6.1%)	374 (8.5%)	138 (2.7%)
Non‐communicable diseases				
Diabetes	407 (12.3%)	649 (16.6%)	765 (17.4%)	404 (7.9%)
Hypertension	681 (20.6%)	937 (24%)	1157 (26.3%)	839 (16.3%)
Chronic kidney disease	84 (2.5%)	143 (3.7%)	205 (4.7%)	87 (1.7%)
Chronic pulmonary disease/asthma	180 (5.5%)	274 (7.0%)	354 (8.0%)	399 (7.8%)
Tuberculosis				
Previous tuberculosis	300 (9.1%)	293 (7.5%)	277 (6.3%)	371 (7.2%)
Current tuberculosis	33 (1.0%)	66 (1.7%)	42 (1.0%)	63 (1.2%)
HIV positive	686 (20.8%)	560 (14.4%)	289 (6.6%)	711 (13.8%)
Prior diagnosed infection	0 (0%)	75 (1.9%)	140 (3.2%)	580 (11.3%)
Vaccination				
Partial^b^	N/A	N/A	26 (0.6%)	269 (5.2%)
Fully^b^	N/A	N/A	127 (2.9%)	1941 (37.7%)
Outcomes within 14 days of diagnosis				
Admission (not severe; not deceased)	272 (8.2%)	428 (11.0%)	456 (10.4%)	322 (6.3%)
Severe admission (not deceased)^c^	Not applicable	131 (3.4%)	189 (4.3%)	45 (0.9%)
death	59 (1.8%)	131 (3.4%)	252 (5.7%)	42 (0.8%)

^a^ Date of diagnoses for cases included in each wave. We included cases diagnosed from 7 days prior to the wave start (deemed to occur when the week on week % change in new admissions exceeded 10%) and for the following 4 weeks, to allow for at least 2 weeks of follow‐up in the most recently diagnosed patients in wave four.

^b^ Fully vaccinated: ≥28 days post vaccination with Janssen/Johnson & Johnson (Ad26.COV2.S) or ≥ 14 days post second dose of Pfizer–BioNTech (BNT162b2); Partially vaccinated: ≥21 days after (first) vaccine dose until meeting criteria for fully vaccinated).

^c^ Admission to an intensive care unit, mechanical ventilation or prescription of oral or intravenous steroids; not reported for wave one as steroids not widely used until after 16 June 2020.

### Comparison of outcomes across waves

Overall, 8.0% (409/5144) of cases were hospitalised or died within 14 days of diagnosis in wave four compared to 16.5% (1918/11,609) across the previous three waves (Table 1). After adjusting for age, sex, comorbidities and subdistrict, there was a substantially reduced hazard of death in wave four compared to wave three (adjusted hazard ratio [aHR] 0.27; 95% confidence interval: 0.19; 0.38) (Table 2). The extent of reduction was attenuated (0.41; 95% CI 0.29; 0.59) when additionally considering prior diagnosed infections and vaccination. Vaccination was strongly protective (aHR for fully vs. not vaccinated 0.20; 95% CI 0.09; 0.43). Wave four was also associated with lower risk of death than waves one and two, which, in turn, were less severe than wave three (aHR [95% CI] for waves one and two vs. wave three were 0.55 [0.40; 0.74] and 0.60 [0.48; 0.74], respectively). The pattern of reduced severity in wave four compared to previous waves was similar for the outcome of severe hospitalisation or death, but for the least specific outcome (i.e. any hospitalisation or death), the risk reduction in wave four versus three was smaller. For all outcomes, the reduced risk of the outcome is attenuated with adjustment for prior diagnosed infection and vaccination. For example, for the outcome of any hospitalisation or death, the risk was lower for wave four versus wave three (aHR 0.72; 95% CI: 0.63; 0.82) and wave two, but greater than wave one (aHR [95%CI] for waves two and one versus wave three: 0.88 [0.80; 0.96] and 0.57 [0.50; 0.66], respectively). Both prior diagnosed COVID‐19 (aHR 0.28; 95% CI 0.19; 0.40) and SARS‐CoV‐2 vaccination were protective (aHR 0.42; 95% CI 0.34; 0.52). Results were very similar when using logistic regression (Table S1).

**TABLE 2:** Associations between different waves and severe COVID‐19 outcomes adjusted for patient characteristics, subdistrict, vaccination, prior diagnosed infection and unascertained prior infections using Cox regression

	Outcome = death not adjusted for vaccination and prior infection		Outcome = death adjusted for vaccination and prior infection		Outcome = severe hospitalisation^a^/death not adjusted for vaccination or prior diagnosed infection		Outcome = severe hospitalisation^a^/death adjusted for vaccination or prior diagnosed infection		Outcome = hospitalisation/death not adjusted for vaccination and prior infection	Outcome = hospitalisation/death adjusted for vaccination and prior infection				
	Adjusted^d^ HR	95% CI	Adjusted HR	95% CI	Adjusted^d^ HR	95% CI	Adjusted HR	95% CI	Adjusted^d^ HR	95% CI	Adjusted HR	95% CI
Male sex (vs. female)	1.44	1.20; 1.73	1.45	1.21; 1.74	1.37	1.19; 1.56	1.37	1.19; 1.57	1.15	1.06; 1.24	1.14	1.06; 1.23	
Age (vs. 20–39 years)													
40–49 years	3.07	1.96; 4.80	3.18	2.03; 4.98	2.32	1.74; 3.10	2.42	1.82; 3.24	1.14	1.00; 1.29	1.17	1.03; 1.33	
50–59 years	7.35	4.88; 11.06	7.64	5.07; 11.52	4.74	3.66; 6.12	4.96	3.82; 6.42	1.90	1.68; 2.15	1.96	1.73; 2.21	
60–69 years	17.17	11.31; 26.05	18.11	11.92; 27.52	8.51	6.52; 11.11	8.85	6.77; 11.58	2.94	2.57; 3.36	3.01	2.63; 3.45	
≥70 years	30.94	20.10; 47.62	34.22	22.22; 52.68	13.56	10.30; 17.85	14.39	10.91; 18.98	4.25	3.70; 4.89	4.40	3.83; 5.06	
Comorbidities (vs. comorbidity absent)													
Diabetes	1.97	1.60; 2.41	1.95	1.58; 2.39	1.76	1.51; 2.05	1.77	1.51; 2.06	2.22	2.02; 2.44	2.26	2.06; 2.48	
Hypertension	1.06	0.87; 1.30	1.05	0.86; 1.28	1.01	0.87; 1.18	1.00	0.86; 1.17	1.11	1.01; 1.21	1.10	1.0; 1.20	
Chronic kidney disease	2.04	1.60; 2.60	2.07	1.63; 2.63	1.73	1.43; 2.09	1.73	1.43; 2.09	1.71	1.52; 1.92	1.70	1.52; 1.91	
Chronic pulmonary disease/asthma	1.24	0.97; 1.59	1.24	0.97; 1.59	1.44	1.20; 1.73	1.46	1.21; 1.75	1.24	1.11; 1.39	1.27	1.13; 1.42	
Previous tuberculosis	1.70	1.24; 2.33	1.68	1.22; 2.29	1.39	1.09; 1.78	1.38	1.08; 1.76	1.17	1.02; 1.34	1.16	1.01; 1.33	
Current tuberculosis	2.24	1.19; 4.21	2.09	1.11; 3.91	3.13	2.10; 4.67	2.99	2.01; 4.47	3.25	2.63; 4.03	3.24	2.63; 3.99	
HIV	1.88	1.36; 2.58	1.92	1.40; 2.64	1.49	1.16; 1.92	1.53	1.19; 1.96	1.58	1.40; 1.78	1.61	1.43; 1.81	
Prior diagnosed infection (vs. None)													
Yes (vs. none)			1.10	0.63; 1.92			0.60	0.37; 0.98			0.28	0.19; 0.40	
Vaccination (vs. None)													
Partial^b^			1.19	0.66; 2.15			1.26	0.81; 1.95			0.89	0.66; 1.20	
Full^b^			0.20	0.09; 0.43			0.23	0.13; 0.39			0.42	0.34; 0.52	
‘Wave period’													
Early wave 1	0.59	0.43; 0.81	0.55	0.40; 0.77					0.60	0.53; 0.69	0.57	0.50; 0.66	
Early wave 2	0.64	0.52; 0.80	0.60	0.48; 0.74	0.74	0.64; 0.85	0.71	0.61; 0.83	0.91	0.83; 0.99	0.88	0.80; 0.96	
Early wave 3	Ref	Ref			Ref	Ref	Ref	Ref	Ref	Ref	Ref	Ref	
Early wave 4	0.27	0.19; 0.38	0.41	0.29; 0.59	0.28	0.22; 0.36	0.43	0.33; 0.55	0.51	0.46; 0.58	0.72	0.63; 0.82	
Early wave 4 accounting for undiagnosed prior infections^c^			0.72				0.75				1.14		

Abbreviations: aHR, adjusted hazard ratio; CI, confidence interval; Ref, Reference.

^a^ Admission to an intensive care unit, mechanical ventilation or prescription of oral or intravenous steroids; not reported for wave one as steroids not widely used until after 16 June 2020.

^b^ Fully vaccinated: ≥28 days post vaccination with Janssen/Johnson & Johnson (Ad26.COV2.S) or ≥ 14 days post second dose of Pfizer–BioNTech (BNT162b2); Partially vaccinated: ≥21 days after (first) vaccine dose until meeting criteria for fully vaccinated).

^c^ We calculated the ‘true hazard ratio’ for the severity of wave four versus wave three using the approach described by Ferguson et al. [18], assuming prior infection reduces risk of death, severe admission or death and admission or death by 80%, 80% and 70%, respectively, and that 15% of prior diagnosed infections were ascertained.

^d^ Adjusted for all variables shown in the table as well as subdistrict/district, but not for vaccination or prior diagnosed infection.

After considering the possible effect of protection against severe outcomes conferred by unascertained prior infection, the reduced risk of severe disease in wave four versus wave three remained but was substantially attenuated with aHR of 0.72 for death and 0.75 for severe hospitalisation or death, respectively, and the risk of any COVID‐19 hospitalisation or death was similar or higher in wave four and wave three (aHR: 1.14). Results were sensitive to the extent of protection assumed to be provided from prior infection and the proportion of prior infections assumed to be ascertained (Table S2). For example, there was no difference in risk of severe hospitalisation or death in wave three versus wave four if the assumed proportion of prior infections detected was reduced from 15% to 12%.

### Protection from vaccination and prior infection in waves three and four

**FIGURE 2:**
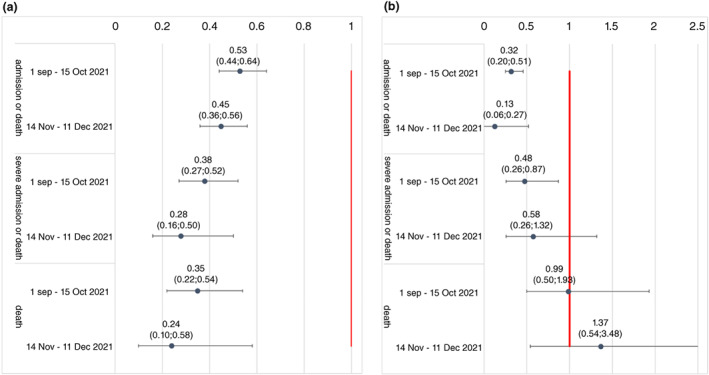
Adjusted hazard ratio for associations between (a) vaccination and (b) prior diagnosed infection and different severe COVID‐19 outcomes adjusted for patient characteristics, subdistrict, vaccination and prior diagnosed infection using Cox regression

Amongst COVID‐19 cases, protection by vaccination against all outcomes was similar in wave four compared to the prior wave (Figure 2). For example, the aHR (95% CI) for protection against death from full vaccination was 0.35 (0.22; 0.54) in late wave three and 0.24 (0.10; 0.58) in wave four. Similarly, the protection conferred by prior diagnosed infection against hospitalisation or death was maintained with aHR (95% CI) of 0.32 (0.20; 0.52) in late wave three and 0.13 (0.06; 0.27) during wave four. The protection conferred by prior infection against other severe COVID‐19 outcomes was difficult to assess due to very small numbers of patients with prior diagnoses experiencing these outcomes.

## DISCUSSION

In this study of Western Cape public sector laboratory‐confirmed COVID‐19 cases, we found a substantial reduction in all severe COVID‐19 outcomes in wave four compared to previous waves with 28% (95% CI: 18%; 37%) reduced risk of any hospitalisation or death and 59% (95% CI: 41%; 71%) reduced risk of death only after adjusting for sociodemographic characteristics, comorbidities and vaccination. Whilst some of this reduction is likely due to retained protection conferred by prior SARS‐CoV‐2 infection against severe disease in Omicron‐infected patients, even when accounting for protection conferred by prior unascertained infections in patients with Omicron, we found a modest reduction in severe hospitalisation or death in wave four versus wave three.

Our findings concur with current literature reporting less severe disease associated with Omicron versus other variants of concern and previous waves [16, 17, 18, 19, 20]. For example, cases with presumed Omicron infection (identified with S‐gene target failure on PCR) were less likely to be admitted to hospital and experience severe outcomes than those with delta infections [18, 19, 21] and fewer admissions with less severe outcomes among those hospitalised have been reported in the fourth wave compared to previous waves in South Africa [16, 17]. To our knowledge, ours is the first study from a setting of high prior seroprevalence to demonstrate less severe disease in wave four after adjusting for both vaccination and prior diagnosed infection and to assess the contribution to protection of a greater proportion of unascertained re‐infections among Omicron cases compared to other variants. Reassuringly, protection against severe disease conferred by prior infection and vaccination was similar in wave four and wave three. Nonetheless, the fact that even after this protection was considered, there was likely reduction of the most severe outcomes in wave four indicates a possible reduction in virulence of Omicron. This is supported by laboratory data from several studies demonstrating possible mechanisms of reduced virulence [6, 7, 8, 14].

The finding of increased prevalence of HIV‐1 in wave four compared to the previous two waves illustrates the importance of adjusting for prior unascertained infections. Poorer urban communities tend to have higher HIV‐1 prevalence and so the pattern of decreasing HIV‐1 prevalence in the second and third waves, with increased prevalence among fourth wave cases, is unlikely to be due to HIV‐1 itself, but because HIV‐1 infection is a proxy for living in a community like Khayelitsha subdistrict, with high SARS‐CoV‐2 seroprevalence by the end of the first wave, which protected against infection in the two subsequent waves, but less so in wave four.

For the less severe outcome of any hospitalisation or death, whilst risk in wave four was less than in wave three, after adjusting for vaccination and prior diagnosed infections, it was similar to that of wave one. This is notable as wave three was driven by the Delta variant, which has been shown to cause more severe disease than ancestral strains [23, 24, 25, 26]. Hospitalisation risk may appear similar to or higher in wave four compared to earlier waves which were due to less transmissible variants. This may simply be because of a higher prevalence of cases during the wave surge together with more widespread testing of asymptomatic hospitalised patients than in previous waves, resulting in more incidental COVID‐19 diagnoses in patients admitted for other conditions. Notwithstanding, the similarity in risk of admission suggests that in the absence of immunity, Omicron could be as severe as the ancestral strain. Irrespective of virulence and disease severity, the sheer number of admissions in patients during an Omicron wave warrants specific planning around appropriate infection prevention and control measures within hospitals whilst minimising adverse impacts on health services for other conditions.

Strengths of our study include complete ascertainment of hospitalisations and deaths in all laboratory‐confirmed COVID‐19 cases across a public sector health service and ability to robustly adjust for comorbidities as well as SARS‐CoV‐2 vaccination. Our analysis also has several limitations. First, we compared outcomes across waves as a proxy for the variant that dominated in each wave and not in patients with genomically confirmed variants. Nonetheless, each wave in South Africa was dominated by a different variant which accounted for >90% of sequenced specimens at the wave peak and using wave as a proxy allowed for a much larger number of cases to be included in the analysis than would have been feasible if limited to genomically confirmed cases. Second, whilst health service pressures which impact disease outcomes are likely to be more similar at ‘equivalent wave periods’, identifying such ‘equivalent periods’ across waves can be challenging. Since we only compared the early part of each wave, our results could be biased if disease severity outcomes differ between the early and later parts of each wave and this difference varies across waves. However, we adjusted for sociodemographic and clinical predictors of disease severity and the results were similar when using slightly different wave periods. Third, most cases in our study would have been tested because of having clinical symptoms, and our findings may have differed if those with asymptomatic infection could have been included. Additionally, as the peak of each wave approached, the Western Cape restricted public sector SARS‐CoV‐2 diagnostic testing to those at greatest risk of severe disease (older people, those with comorbidities or needing admission) which could have impacted on comparison of disease outcomes across waves. However, since we only included the early part of each wave in this analysis, there were only 4 days included (all in the third wave period—19–23 June 2021) when testing was restricted, and the results did not differ when we excluded these 4 days from the analysis. Fourth, adjustment for comorbidities was limited to those algorithmically identified in the WCPHDC and does not include undiagnosed comorbidities and other important risk factors for poor COVID‐19 outcomes such as obesity. Fifth, prior diagnosed infections substantially under ascertain all prior infections, and whilst we addressed this by determining the likely impact of undiagnosed infections in wave four, this is based on assumptions. Furthermore, due to the high prevalence of prior infections in our population, our estimates of vaccine protection against severe disease among COVID‐19 cases may underestimate the full protection that vaccines would provide in an infection‐naive population. Sixth, we could not distinguish between admissions and deaths where the diagnosis of COVID‐19 may have been incidental or contributory rather than causal. However, our main analysis focused on mortality and we found stronger protection of both wave four and vaccination against the more severe outcomes (severe hospitalisation and/or death), suggesting that results are robust despite misclassification of admissions with incidental COVID‐19. Nonetheless, a different clinical profile of hospitalised and deceased patients with COVID‐19 has been reported, with less COVID‐19 pneumonia and a greater proportion of patients with severe comorbidities where COVID‐19 may be contributory, but not causing typical respiratory presentation [17]. We may, therefore, be underestimating the reduction in risk of hospitalisation and death due to COVID‐19 pneumonia specifically in the Omicron wave. Seventh, we excluded children as the effects of Omicron on disease severity may differ in children compared to adults, the role of COVID‐19 as a cause of pathology in a child admitted with respiratory illness with several different viruses present is unclear, and because we could not assess differences in the more severe outcomes between waves in children as these were so uncommon. Finally, outcomes were limited to 14 days post diagnosis to allow for equivalent follow‐up in the most recent versus previous waves and so we could not compare outcomes beyond 14 days. However, in previous waves, only 2.9% of hospitalisations occurred beyond this period and only 2.6% of deaths had not been hospitalised or deceased within 2 weeks of diagnosis.

In conclusion, we found substantially reduced disease severity amongst diagnosed COVID‐19 cases in the Omicron‐driven fourth wave compared to previous waves. Whilst this appears to be largely due to retained protection against severe outcomes conferred by prior infection and vaccination, our data suggest that there may be a modest reduction in severe outcomes due to intrinsically reduced virulence of Omicron.

## CONFLICT OF INTEREST

All authors declare that they have no conflicts of interest.

## DISCLOSURE

The funders had no role in the study design, data collection, data analysis, data interpretation or writing of this report. The opinions, findings and conclusions expressed in this manuscript reflect those of the authors alone. For the purposes of open access, the author has applied a CC‐BY public copyright to any author accepted version arising from this submission.

## Supporting information


**Table S1** Associations between different waves and severe COVID‐19 outcomes adjusted for patient characteristics, subdistrict, vaccination and prior diagnosed infection using logistic regression
**Table S2** Range of possible values for the true hazard ratio for the association between wave four (vs. wave three) and each COVID‐19 outcome for different assumptions of the protection that prior infection provides against the outcome and the proportion of reinfections detected among cases diagnosed during the fourth wave
