# Supplementary material for: Outcomes of laboratory‐confirmed SARS‐CoV‐2 infection in the Omicron‐driven fourth wave compared with previous waves in the Western Cape Province, South Africa
Source: Trop Med Int Health. 2022 May 10;27(6):564–73. doi: 10.1111/tmi.13752 (PMC9115442; doi:10.1111/tmi.13752)
Supplement: Supplementary file 1 — Table S1 Associations between different waves and severe COVID‐19 outcomes adjusted for patient characteristics, subdistrict, vaccination and prior diagnosed infection using logistic regression Table S2 Range of possible values for the true hazard ratio for the association between wave four (vs. wave three) and each COVID‐19 outcome for different assumptions of the protection that prior infection provides against the outcome and the proportion of reinfections detected among cases diagnosed during the fourth wave [file TMI-27-564-s001.docx]

**Supplementary Table 1:** Associations between different waves and severe COVID-19 outcomes adjusted for patient characteristics, sub-district, vaccination and prior diagnosed infection using logistic regression.

|  | **Outcome = death not adjusted for vaccination and prior infection** | | **Outcome = death adjusted for vaccination and prior infection** | |  | **Outcome = severe hospitalization^#^/death not adjusted for vaccination or prior diagnosed infection** | | **Outcome = severe hospitalization^#^/death adjusted for vaccination or  prior diagnosed infection** | |  | **Outcome = hospitalization/death not adjusted for vaccination and prior infection** | | **Outcome = hospitalization/death adjusted for vaccination and prior infection** | |
| --- | --- | --- | --- | --- | --- | --- | --- | --- | --- | --- | --- | --- | --- | --- |
|  | **Adjusted^^^ OR** | **95% CI** | **Adjusted OR** | **95% CI** |  | **Adjusted^^^ OR** | **95% CI** | **Adjusted OR** | **95% CI** |  | **Adjusted^^^ OR** | **95% CI** | **Adjusted OR** | **95% CI** |
| **Male sex (vs. female)** | 1.53 | 1.26; 1.88 | 1.56 | 1.27; 1.90 |  | 1.47 | 1.25; 1.73 | 1.47 | 1.24; 1.73 |  | 1.21 | 1.10; 1.34 | 1.20 | 1.08; 1.33 |
| **Age (vs. 20-39 years)** |  |  |  |  |  |  |  |  |  |  |  |  |  |  |
| **40-49 years** | 2.99 | 1.91; 4.71 | 3.09 | 1.96; 4.86 |  | 2.30 | 1.71; 3.10 | 2.40 | 1.78; 3.24 |  | 1.08 | 0.93; 1.25 | 1.12 | 0.96; 1.29 |
| **50-59 years** | 7.18 | 4.73; 10.89 | 7.44 | 4.89; 11.31 |  | 4.84 | 3.69; 6.35 | 5.07 | 3.85; 6.67 |  | 1.92 | 1.66; 2.22 | 1.99 | 1.72; 2.31 |
| **60-69 years** | 17.43 | 11.36; 26.76 | 18.42 | 11.98; 28.31 |  | 9.35 | 6.99; 12.49 | 9.76 | 7.28; 13.08 |  | 3.38 | 2.85; 4.01 | 3.52 | 2.96; 4.19 |
| **≥70 years** | 35.14 | 22.57; 54.72 | 39.44 | 25.29; 61.50 |  | 17.43 | 12.86; 23.63 | 18.79 | 13.80; 25.59 |  | 6.53 | 5.39; 7.91 | 6.90 | 5.67; 8.40 |
| **Comorbidities (vs. comorbidity absent)** | |  |  |  |  |  |  |  |  |  |  |  |  |  |
| **diabetes** | 2.13 | 1.71; 2.67 | 2.12 | 1.69; 2.66 |  | 2.02 | 1.68; 2.43 | 2.03 | 1.68; 2.45 |  | 3.06 | 2.70; 3.47 | 3.16 | 2.79; 3.59 |
| **hypertension** | 1.07 | 0.86; 1.34 | 1.06 | 0.85; 1.32 |  | 1.00 | 0.83; 1.21 | 0.99 | 0.82; 1.19 |  | 1.12 | 0.99; 1.26 | 1.11 | 0.98; 1.25 |
| **chronic kidney disease** | 2.33 | 1.75; 3.09 | 2.38 | 1.79; 3.16 |  | 2.20 | 1.69; 2.87 | 2.19 | 1.68; 2.85 |  | 3.04 | 2.42; 3.82 | 3.01 | 2.39; 3.80 |
| **chronic pulmonary disease / asthma** | 1.32 | 0.99; 1.76 | 1.33 | 1.0; 1.78 |  | 1.64 | 1.29; 2.08 | 1.67 | 1.31; 2.12 |  | 1.41 | 1.19; 1.67 | 1.46 | 1.23; 1.73 |
| **previous tuberculosis** | 1.73 | 1.23; 2.43 | 1.70 | 1.21; 2.40 |  | 1.45 | 1.09; 1.94 | 1.44 | 1.08; 1.92 |  | 1.23 | 1.03; 1.46 | 1.20 | 1.01; 1.44 |
| **current tuberculosis** | 2.48 | 1.21; 5.10 | 2.30 | 1.12; 4.74 |  | 4.06 | 2.42; 6.82 | 3.82 | 2.28; 6.39 |  | 5.02 | 3.65; 6.89 | 4.92 | 3.58; 6.75 |
| **HIV** | 1.98 | 1.41; 2.80 | 2.04 | 1.45; 2.87 |  | 1.60 | 1.21; 2.13 | 1.64 | 1.24; 2.17 |  | 1.74 | 1.50; 2.01 | 1.77 | 1.53; 2.05 |
| **Prior diagnosed infection vs. none** |  |  |  |  |  |  |  |  |  |  |  |  |  |  |
| **Yes (vs none)** |  |  | 1.14 | 0.61; 2.11 |  |  |  | 0.59 | 0.34; 1.03 |  |  |  | 0.22 | 0.14; 0.34 |
| **Vaccination vs. none** |  |  |  |  |  |  |  |  |  |  |  |  |  |  |
| **partial^*^** |  |  | 1.25 | 0.60; 2.61 |  |  |  | 1.43 | 0.79; 2.59 |  |  |  | 0.87 | 0.57; 1.32 |
| **full^*^** |  |  | 0.19 | 0.08; 0.42 |  |  |  | 0.21 | 0.12; 0.37 |  |  |  | 0.36 | 0.28; 0.46 |
| **"Wave period"** |  |  |  |  |  |  |  |  |  |  |  |  |  |  |
| **early wave 1** | 0.56 | 0.39; 0.79 | 0.52 | 0.37; 0.74 |  | Not reported^#^ | | Not reported^#^ | |  | 0.50 | 0.43; 0.59 | 0.48 | 0.40; 0.56 |
| **early wave 2** | 0.61 | 0.48; 0.77 | 0.56 | 0.44; 0.71 |  | 0.69 | 0.57; 0.82 | 0.66 | 0.55; 0.79 |  | 0.86 | 0.76; 0.98 | 0.84 | 0.74; 0.95 |
| **early wave 3** |  |  |  |  |  |  |  |  |  |  |  |  |  |  |
| **early wave 4** | 0.25 | 0.17; 0.36 | 0.38 | 0.26; 0.55 |  | 0.24 | 0.19; 0.31 | 0.37 | 0.28; 0.50 |  | 0.43 | 0.37; 0.50 | 0.65 | 0.55; 0.76 |
| *Fully vaccinated: ≥28 days post-vaccination with Janssen/Johnson & Johnson (Ad26.COV2.S) or ≥14 days post second dose of Pfizer–BioNTech (BNT162b2); Partially vaccinated: ≥21 days after (first) vaccine dose until meeting criteria for fully vaccinated); ^#^Admission to an intensive care unit, mechanical ventilation or prescription of oral or intravenous steroids; not reported for wave 1 as steroids not widely used until after 16 June 2020. ^^^Adjusted for all variables shown in the table as well as subdistrict/district, but not for vaccination or prior diagnosed infection; aOR = adjusted Odds Ratio; CI = Confidence Interval | | | | | | | | | | | | | | |

**Supplementary Table 2:** Range of possible values for the true hazard ratio for the association between wave four (vs. wave three) and each COVID-19 outcome for different assumptions of the protection that prior infection provides against the outcome and the proportion of reinfections detected among cases diagnosed during the fourth wave.

|  |  | **Outcome = death;  Observed aHR: 0.41 (95%CI: 0.29; 0.59)** | | | | |  | **Outcome = severe admission/death; Observed aHR: 0.43 (95%CI: 0.33; 0.55)** | | | | |  | **Outcome = admission or death; Observed aOR: 0.72 (95%CI:0.63; 0.82)** | | | | |
| --- | --- | --- | --- | --- | --- | --- | --- | --- | --- | --- | --- | --- | --- | --- | --- | --- | --- | --- |
| **Relative risk of outcome in those with vs. without prior infection** |  | **0.10** | **0.15** | **0.20** | **0.25** | **0.30** |  | **0.10** | **0.15** | **0.20** | **0.25** | **0.30** |  | **0.20** | **0.25** | **0.30** | **0.35** | **0.40** |
| **Proportion of reinfections detected** | **0.12** | 1.27 | 1.11 | 0.99 | 0.89 | 0.82 |  | 1.33 | 1.16 | 1.04 | 0.94 | 0.86 |  | 1.74 | 1.57 | 1.43 | 1.32 | 1.23 |
| **Proportion of reinfections detected** | **0.15** | 0.81 | 0.76 | 0.72 | 0.68 | 0.65 |  | 0.85 | 0.80 | 0.75 | 0.71 | 0.68 |  | 1.26 | 1.20 | 1.14 | 1.09 | 1.04 |
| **Proportion of reinfections detected** | **0.20** | 0.61 | 0.59 | 0.58 | 0.56 | 0.55 |  | 0.64 | 0.62 | 0.60 | 0.59 | 0.57 |  | 1.01 | 0.98 | 0.96 | 0.93 | 0.91 |
| **Proportion of reinfections detected** | **0.25** | 0.54 | 0.53 | 0.52 | 0.51 | 0.50 |  | 0.57 | 0.55 | 0.54 | 0.54 | 0.53 |  | 0.91 | 0.90 | 0.88 | 0.87 | 0.85 |
| **Observed reinfections during wave4  (proxy for omicron)** | **0.11** | **0.11** | **0.11** | **0.11** | **0.11** | **0.11** |  | **0.11** | **0.11** | **0.11** | **0.11** | **0.11** |  | **0.11** | **0.11** | **0.11** | **0.11** | **0.11** |

aHR = adjusted Hazard Ratio; CI = confidence interval
